# A Subgroup of Latently *Mycobacterium tuberculosis* Infected Individuals Is Characterized by Consistently Elevated IgA Responses to Several Mycobacterial Antigens

**DOI:** 10.1155/2015/364758

**Published:** 2015-08-10

**Authors:** Ralf Baumann, Susanne Kaempfer, Novel N. Chegou, Wulf Oehlmann, Ralf Spallek, André G. Loxton, Paul D. van Helden, Gillian F. Black, Mahavir Singh, Gerhard Walzl

**Affiliations:** ^1^DST/NRF Centre of Excellence for Biomedical TB Research and MRC Centre for TB Research, Division of Molecular Biology and Human Genetics, Department of Biomedical Sciences, Faculty of Medicine and Health Sciences, Stellenbosch University, P.O. Box 19063, Tygerberg 7505, South Africa; ^2^Lionex Diagnostics and Therapeutics, Salzdahlumer Straße 196, 38126 Braunschweig, Germany

## Abstract

Elevated antibody responses to *Mycobacterium tuberculosis* antigens in individuals with latent infection (LTBI) have previously been linked to an increased risk for progression to active disease. Studies in the field focussed mainly on IgG antibodies. In the present study, IgA and/or IgG responses to the mycobacterial protein antigens AlaDH, NarL, 19 kDa, PstS3, and MPT83 were determined in a blinded fashion in sera from 53 LTBI controls, 14 healthy controls, and 42 active TB subjects. Among controls, we found that elevated IgA levels against all investigated antigens were not randomly distributed but concentrated on a subgroup of <30%—with particular high levels in a small subgroup of ~5% comprising one progressor to active TB. Based on a specificity of 100%, anti-NarL IgA antibodies achieved with 78.6% sensitivity the highest accuracy for the detection of active TB compared to healthy controls. In conclusion, the consistently elevated IgA levels in a subgroup of controls suggest higher mycobacterial load, a risk factor for progression to active TB, and together with high IgG levels may have prognostic potential and should be investigated in future large scale studies. The novel antigen NarL may also be promising for the antibody-based diagnosis of active TB cases.

## 1. Introduction

Approximately one third of the world's population has latent infection with* Mycobacterium tuberculosis* [1]. Latent* M. tuberculosis* infection (LTBI) represents a considerable reservoir of future active disease and contagion. Risk factors include co-infection with human immunodeficiency virus (HIV), diabetes mellitus, low body weight, old age, or use of immunosuppressive medications. In immunocompetent individuals, the annual risk of progression is estimated to be greatest in the first 1 or 2 years after infection.

Preventing LTBI individuals from reactivation (before they become symptomatic and infectious) may constitute a major step towards the elimination of TB. Therefore, the revised global plan to stop TB (2011–15) [2] has set 2015 as the goal for point-of-care tests that can be used for the accurate detection of preclinical TB.

Bacterial load is associated with disease risk, and antibody levels against* M. tuberculosis *components may be biomarkers for load [3] as well as disease risk [4–6]. Stratification of TB suspects into groups of absent, low (smear-negative tuberculosis), and high (smear-positive tuberculosis) bacterial burden showed that antibody levels correlated with bacillary burden [4]. In the same study, data of the macaque model, which reproduces key features of latent TB in humans, showed that infection outcome was reflected by the antibody response in the latent infection group [4], thereby confirming previous animal studies [7–9]. Although studies were small, increased specific antibody levels during the LTBI stage in humans also characterized progressors [5, 6]. Antibody-based tests for the diagnosis of active TB disease are often criticized for their lack of specificity in TB endemic regions [10], which is due to a high background prevalence of LTBI [11]. Clearly, further research is needed to elucidate whether* M. tuberculosis* specific antibody tests can determine active TB and cases at risk for progression and whether lack of specificity of antibody-based TB tests will turn out to be due to a high risk for an early stage of progression to active TB.

The low frequency of reactivation in immunocompetent LTBI individuals poses a challenge for the discovery of prognostic markers. Therefore, we chose to investigate the distribution of serologic responses, as a nonrandom distribution may point to a subgroup with higher bacterial load and an increased risk for future progression to disease. In a South African TB endemic population, we evaluated the serodiagnostic reactivity of L-alanine dehydrogenase (AlaDH) (Rv2780), nitrate/nitrite response transcriptional regulator NarL (Rv0844c), periplasmic phosphate-binding lipoprotein PstS3 (Rv0928), 19 kDa lipoprotein antigen precursor LpqH (Rv3763), and lipoprotein MPT83 (Rv2873). IgG responses to each of the two surface-exposed lipoproteins, 19 kDa and MPT83, are predominantly recognized in active TB sera and not in non-TB disease (NTBD) sera [4]. The 19 kDa antigen promotes binding to host cells and phagocytosis of mycobacteria [12], inhibits IFN-*γ*-induced killing of mycobacteria by macrophages [13], and induces macrophage apoptosis [14]. MPT83 elicits T cell proliferation of the majority of TB patients and is being considered as future subunit vaccine candidate [15]. The third lipoprotein,* M. tuberculosis* PstS3, which is involved in active transport of inorganic phosphate across the membrane (import), has not been investigated yet in subjects with LTBI for the serodiagnosis of* M. tuberculosis *[16]. However, PstS3 generates IFN-*γ*-producing cells in a more potent manner than the closely related 38 kDa (PstS1) [17], a major* M. tuberculosis* antigen [4, 18, 19]. The in TB serodiagnostics newly investigated protein antigen NarL is a putative nitrate response regulator involved in the regulation of anaerobic metabolism [20] and is part of the membrane fraction of* M. tuberculosis* [21]. IgG responses to the culture filtrate (and membrane) protein AlaDH are unable to distinguish untreated TB patients and controls in endemic settings [22]. AlaDH is present in* M. tuberculosis* but not in the vaccine strain* Mycobacterium bovis* BCG [21, 23]. It may play a role in cell wall synthesis as L-alanine is an important constituent of the peptidoglycan layer.

We focused on IgA antibodies because* M. tuberculosis*-specific IgA antibodies discriminated better than IgG antibodies between active TB and TB endemic controls in Africa [24] as well as between healthy close contacts of pulmonary TB patients and healthy individuals without such contact [25]. Moreover, IgA production was reported to be highly T-cell dependent [26], and a protective role for IgA was suggested in several murine models of mycobacterial infection, for example, [27].

## 2. Materials and Methods

### 2.1. Study Population

Sera utilized to probe antibody assays were from a retrospective serum bank collected from individuals in an epidemiological field site in metropolitan Cape Town in South Africa with a population of whom 99.7% are of mixed race. The incidence of new smear-positive TB in this community was 341/100 000 population in 2002 and the majority of people harbours latent infection [28]. In the study community, BCG vaccination (Danish strain, 1331, Statens Serum Institute, Copenhagen, Denmark) is routinely administered at birth since 1971. The study was approved by the Ethics Committee of the Faculty of Health Sciences at the Stellenbosch University and written informed consent was obtained from all participants or their legal guardians in the case of children.

*Community Control Subjects*. Inclusion criteria for all healthy control participants enrolled into the study were residence in the described community, absence of clinical signs of TB or other diseases, absence of prior TB, HIV negativity, and no pregnancy. Several parameters were employed to determine TB infection status: the Mantoux skin test, two different and independent commercial interferon-*γ* release assays (IGRAs) [the QuantiFERON TB Gold in Tube (QFT) (Qiagen, Hilden, Germany, Australia) and T-SPOT.*TB* (Oxford Immunotec, Abingdon, UK)], chest X-ray (CXR), and sputum AFB staining. We used >15 mm as cut off for Mantoux test positivity [29] and alternatively >5 mm [30] as described in more detail in the results section. Two IGRAs were used, as rates of positive results have been reported to differ between T-SPOT.TB and QuantiFERON-TB Gold (e.g. [31, 32]). Sixty-four consecutively recruited recent household contacts of active TB patients were part of a larger household contact study, and because the non-LTBI individuals constituted fewer than 20% of the contacts, three additional community controls with no known previous TB exposure were added and underwent the same investigations as the household contacts except the IGRA assays. The majority of LTBI subjects were followed up for the development of active TB disease within 2 years after their first recruitment, and one progressor (after 3 months) was identified according to hospital records. 

*TB Patients*. Forty-two HIV-negative, Ziehl-Neelsen sputum smear-positive and BACTEC sputum culture-positive active pulmonary TB patients with no known multidrug resistance that were part of the same larger study as the controls, of which results have been published recently [33, 34], were included. Seven TB patients were excluded due to HIV-seropositivity (*n* = 1), NTM infection (*n* = 2), or concomitant illness, such as diabetes mellitus (*n* = 4). All TB patients were self-reporting, untreated cases, and all except 2 had a first episode of active TB.

### 2.2. Serum Preparation

Blood samples of control subjects and TB patients (at diagnosis prior to initiation of treatment) were taken. After transport of the blood samples to the laboratory (within 2 h of collection and at ambient conditions), serum was separated by centrifugation (1250 ×g for 7 min) and stored in aliquots at −80°C until use.

### 2.3. Antigen Cloning, Protein Expression, and Purification

The production of the functional* M. tuberculosis* L-alanine dehydrogenase (AlaDH) in the heat-induced strain* Escherichia (E.) coli* CAG629 (pMSK12) has been described previously [35]. For cloning, the genes of the remaining 4 protein antigens (Table 1) were amplified by PCR using primers with integrated restriction sites allowing the site-directed insertion of cleaved PCR-products into pET vectors (Novagen). All four genes were fused to sequence coding for 6-fold His-tag (Table 1). The genes were expressed in* E. coli* BL21(DE3) or in case of* pstS3* in* E. coli* Rosetta (DE3). The antigens were purified using standard chromatographic methods (affinity chromatography, ion exchange chromatography, size exclusion chromatography). Insoluble antigens were solubilized (refolded) from denaturating conditions (8 M urea) into buffers free of choatropic reagents. Further details regarding the protein purification are described in the supplements (see Supplementary Material available online at http://dx.doi.org/10.1155/2015/364758).

### 2.4. Enzyme-Linked Immunosorbent Assay

Microtiter plates were coated with* M. tuberculosis* antigens and serologic antibody responses were determined using standard procedures as described in the supplements. Laboratory personnel performing the serodiagnosis assays were blinded to the clinical status of the patients or the controls. Subsequent record reviews were done by clinical staff to classify the individuals into clinical groups without knowledge of the serologic response phenotype.

### 2.5. Statistical Analysis

GraphPad Prism (Graph Pad, San Diego, CA, USA) was used to create graphs and statistical analyses were performed using Medcalc software (Kagi, Berkeley, CA). The *t*-test for independent samples or the Mann-Whitney test was used for the statistical comparison of 2 groups depending on the fact whether or not the data were normally distributed. Given the aim to develop a diagnostic test for TB with a specificity level above at least 90%, the experiments were analyzed by using specificity levels of ≥ 90%, as was done by other investigators [36, 37]. The Spearman rank test was used for correlation analyses. Generally, a two-tailed *P* value of *P* ≤ 0.05 was considered significant.

## 3. Results

### 3.1. Classification of Study Participants according to Their* Mycobacterium tuberculosis* Infection Status

Sixty-four healthy household contacts of recently (within past 2 months) diagnosed active pulmonary TB patients underwent TST and 2 commercial IGRA tests, QFT, and T-SPOT.TB. Fifty-three household contacts were classified as LTBI due to positivity in TST (induration ≥ 15 mm) [29], QFT and/or T-SPOT.TB (*n* = 53; age range: 15–59 years; 58.2% females). The remaining 11 household contacts were classified as healthy controls (HC). As noninfected control individuals were rare in the described TB endemic settings [28], we added 3 healthy nonhousehold contacts (community controls with no known previous TB exposure) with TST-indurations of 0 mm, normal chest X-rays and AFB-negative-assisted sputum samples to the healthy control group in order to increase the statistical power (*n* = 14; age range: 10.7–55.2 years; 71.4% females). The Mantoux induration > 15 mm criterion was used for the definition of LTBI, as a large-scale study performed in a rural African population showed that the local environmental mycobacterial exposure is reflected in Mantoux indurations that cluster around 10 mm, whereas the* M. tuberculosis* exposures are reflected by indurations with a mode at 15–17 mm or larger [29]. Still, the exact definition of the LTBI status is hampered by the lack of a gold standard. According to Centers for Disease Control and Prevention (CDC) criteria Mantoux induration > 5 mm of recent TB household contacts can be considered as LTBI [30], and we alternatively considered this cut off for defining a positive tuberculin reaction: of the fourteen members of the original HC group, two subjects had Mantoux indurations between 5 mm and 15 mm, and both individuals had negative IGRA results. Therefore, in an alternative grouping of the controls, we transferred the 2 IGRA-negative individuals with Mantoux indurations between 5 and 15 mm from the HC group (HC^*∗*^: *n* = 12) into the LTBI group (LTBI^*∗*^: *n* = 55). The 42 smear-positive active pulmonary TB patients (age range: 18–55 years; 40.5% females) included in this study were recruited from the same community as the control participants.

### 3.2. Profile of Specific IgA and/or IgG Antibodies in an Endemic Setting

**Figure 1:**
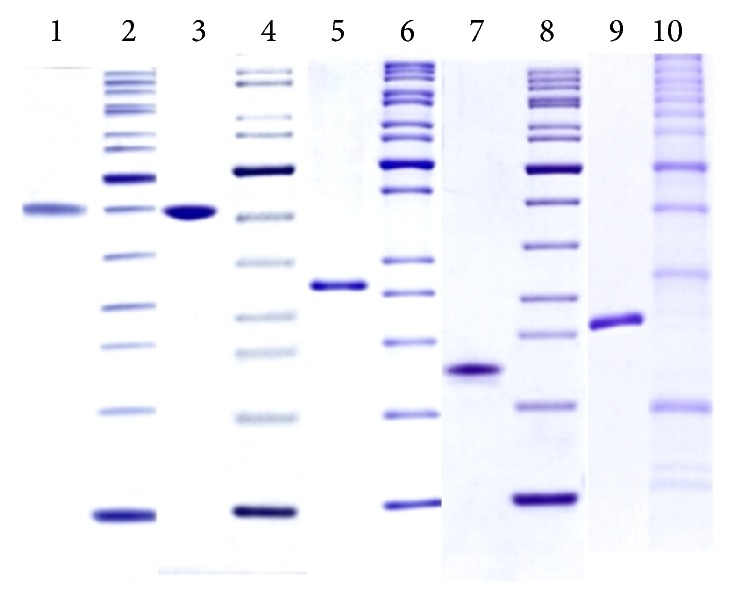
Quality control SDS-PAGE analysis (reduced) of the highly purified recombinant antigens (1 *μ*g per lane).* Lane 1*: PstS3,* Lane 3*: AlaDH,* Lane 5*: MPT83,* Lane 7*: 19 kDa,* Lane 9*: NarL,* Lanes* 2, 4, 6, 8, and 10: molecular weight ladders (each corresponding to the antigen on the left side),* Lanes* 1–4: 15% Laemmli gels, and* Lanes* 5–10: 12% Laemmli gels. All stained with Coomassie brilliant blue R250.

The 5 protein antigens NarL, AlaDH, 19 kDa, PstS3, and MPT83 (Table 1) were cloned and expressed in* E. coli* and purified using standard chromatographic methods. A Coomassie Brilliant Blue R-250 stained SDS-PAGE analysis of the 5 proteins is shown in Figure 1.

**Figure 2:**
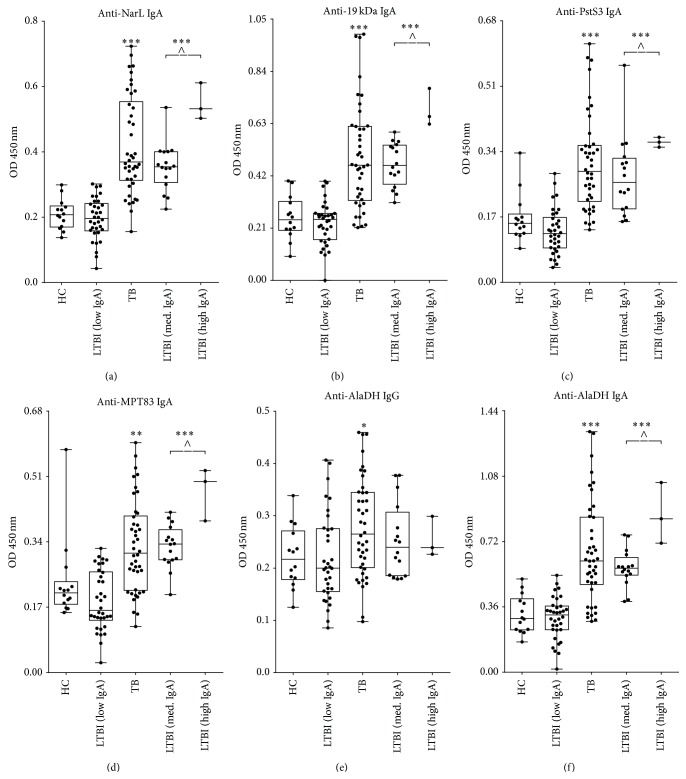
Box-and-whisker plots including individual points for the comparison of 109 TB or non-TB specimens showing the optical density (OD) values of the following selected antigens: anti-NarL IgA (a), anti-19 kDa IgA (b), anti-PstS3 IgA (c), anti-MPT83 IgA (d), anti-AlaDH IgG (e), and anti-AlaDH IgA (f). Values are shown for sera from 14 healthy controls (HC), 42 TB patients, and 3 different LTBI groups. The mean ranks of IgA signals against the 5 antigens investigated were used to group the LTBI group into the top 3 group [LTBI (high IgA); comprising the progressor to active TB disease], the following 16 mean ranks [LTBI (medium IgA)] and the remaining LTBI group [LTBI (low IgA)]. The symbol *∗* depicted above either the TB patients or the LTBI (high and medium IgA) group shows a significant difference of this group compared to healthy controls. A *P* value of ≤ 0.05 was judged significant and levels of significance were indicated as follows: _ _^*∗*^ *P* = 0.01–0.05, _ _^*∗∗*^ *P* = 0.001–0.01, ^*∗∗∗*^ *P* < 0.001.

The 109 serum samples derived from 42 pulmonary TB patients, 53 LTBI controls, and 14 non-LTBI controls were tested in a blinded fashion for IgA responses specific to the 5 proteins as well as for IgG responses to AlaDH. Among controls, we found that elevated IgA levels against the investigated 5 antigens were not randomly distributed but concentrated on a subgroup of 28.4% (*n* = 19)—with particular high levels in a subgroup of 4.5% (*n* = 3), which comprised the progressor from latent infection to active TB. To graphically distinguish between those LTBI individuals with negative serum data (more than 70% of the controls) and those with moderately elevated and highly elevated serology, we used the mean ranks of IgA signals against the 5 antigens investigated to divide the LTBI subjects into the 3 subgroups LTBI (low IgA), LTBI (medium IgA) and LTBI (high IgA) (Figure 2). Furthermore, we statistically compared the non-LTBI controls with either the active TB patients or the LTBI subgroup with elevated IgA signals comprising the 2 subgroups LTBI (medium IgA) and LTBI (high IgA) (Table 2). Based on a cut-off referring to the 92.9-percentile of the healthy non-LTBI controls [92.9% (95% CI, 66.1–99.8%) specificity], the IgA response against the novel protein antigen NarL achieved with 81% (95% CI, 65.9–91.4%) the highest sensitivity for the detection of active TB patients, followed by anti-AlaDH IgA with 76.2% (95% CI, 60.5–87.9%) sensitivity and anti-19 kDa IgA with 64.3% (95% CI, 48.0–78.4%) sensitivity (Table 2). Moreover, based on a specificity of 100% (95% CI, 76.8–100%), anti-NarL IgA and anti-AlaDH IgA both detected 84.2% (95% CI, 60.4–96.6%) of the LTBI subgroup with elevated IgA signals, followed by anti-19 kDa IgA with 78.9% (95% CI, 54.4–93.9%) sensitivity (Table 2). Anti-NarL IgA, anti-19 kDa IgA, and anti-AlaDH IgA detected the LTBI (high IgA) subgroup, which comprised the progressor to active TB, with a distinct signal-to-noise ratio compared to the non-LTBI community controls (Figures 2(a), 2(b), and 2(f)). In contrast, the LTBI (high IgA) subgroup was indistinguishable from the non-LTBI community controls when using the IgG response to AlaDH (Figure 2(e)). Very similar results to those shown in Table 2 were obtained when using the alternative groups HC^*∗*^ and LTBI^*∗*^ (Table S1).

**Figure 3:**
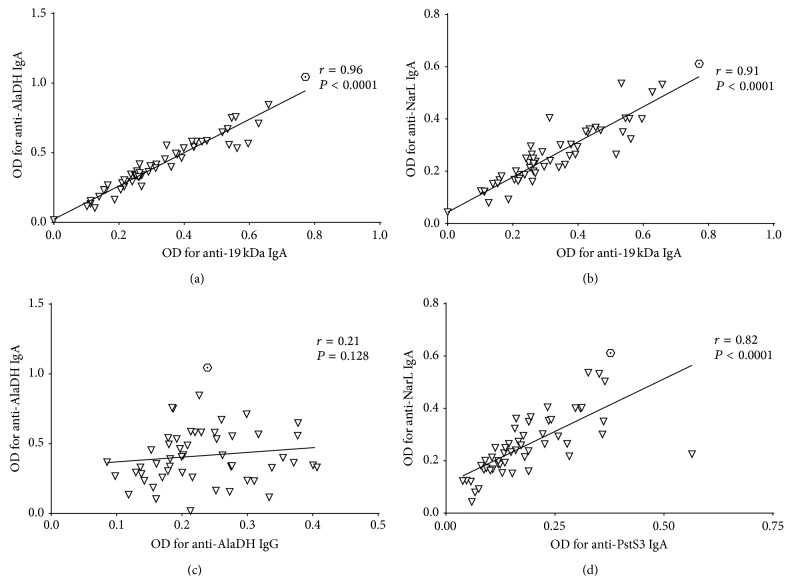
Correlations of IgA antibody levels in serum of latently* M. tuberculosis* infected individuals. Scatter graphs showing the relations between anti-AlaDH IgA and anti-19 kDa IgA (a), anti-NarL IgA and anti-19 kDa IgA (b) and anti-AlaDH IgA and anti-AlaDH IgG (c) as well as anti-NarL IgA and anti-PstS3 IgA (d) using the optical density (OD) values in diluted sera (*n* = 53). The correlation coefficient *r* and the *P* value were calculated using the Spearman rank test. The hexagon with a dot in its center characterizes the progressor to active TB, whereas all other latently* M. tuberculosis* infected individuals are characterized by the symbol ∇.

When testing for correlations between the antibody OD values in LTBI sera (*n* = 53) (Table 3 and Figure 3), we found strong correlations between the IgA responses and the 5 protein antigens [strongest correlation between anti-AlaDH IgA and anti-19 kDa IgA: Spearman's *r* = 0.96 (95% CI, 0.93–0.98); *P* < 0.0001 (Figure 3(a)); weakest correlation between anti-NarL IgA and anti-PstS3 IgA: *r* = 0.82 (95% CI, 0.71–0.89); *P* < 0.0001 (Figure 3(d))]. In contrast, we found no or negligible correlations of any of the IgA responses with the exemplarily tested IgG response to AlaDH [e.g., no correlation between anti-AlaDH IgA and anti-AlaDH IgG: *r* = 0.21 (95% CI, −0.06–0.46); *P* = 0.128 (Figure 3(c))] (Table 3). Very similar results to those shown in Table 3 were obtained when using the alternative group LTBI^*∗*^ (Table S2).

## 4. Discussion

In recent years it became increasingly clear that antibody-based diagnostics of active TB (often developed and tested in non-TB endemic countries) performed poorly in TB endemic settings [10] due to high background signals of antibodies in LTBI individuals [11]. In our study we also found that the presence of LTBI affected identification of active TB by serology. In particular, we found that among controls elevated IgA levels against the investigated 5 antigens were not randomly distributed but concentrated on a subgroup of <30%—with particular high levels in a small subgroup of ~5%. The consistently elevated IgA levels in a subgroup of controls suggest higher mycobacterial load, a risk factor for progression to active TB. Whether high IgA and/or IgG levels have prognostic potential should be investigated in future large scale studies.

In several previous reports it has indeed been suggested, directly or indirectly, that progression to active TB may be predicted by increased specific antibodies levels. Certain TB-specific antibody responses decline significantly during successful therapy (and the time thereafter) when both bacterial load and risk of disease also decline [38, 39]. In inactive TB (defined by a positive response to the TST, negative sputum cultures, and abnormal but stable CXR findings), a special form of latent infection known to have increased risk of active TB, elevated specific antibody responses have been reported [40, 41]. Several independent studies showed that antibody responses to mycobacterial proteins were detectable months to years prior to the diagnosis of TB in persons infected with HIV, for example, [8], again suggesting that* in vivo M. tuberculosis *replication may begin long before progression to active TB becomes clinically detectable. Specific antibacterial antibodies have been shown to be present in sera obtained from* M. tuberculosis* H37Rv aerosol-infected rabbits, and guinea pigs both with preclinical TB [7, 8]. In mouse models of* M. avium *infection, susceptibility to infection correlated with increased synthesis of specific anti-bacterial antibodies [9]. Integration of macaque and human proteome-scale antibody profiling data revealed dynamic characteristics of the antibody response in relation to bacillary burden and infection outcome [4]. Two individuals with elevated specific IgG responses originally categorized as LTBI were subsequently diagnosed to have culture-confirmed active TB within a few weeks of the serologic testing [6]. Moreover, among apparently healthy professional contacts of TB patients (or pathological specimens thereof), elevated specific IgG and/or IgM responses to mycobacterial antigens were determined in a subgroup of 9 individuals, of whom 4 (44.4%) developed active TB within one year after serological testing [5].

Clearly, future large scale, long term prospective studies in different TB endemic areas are needed to further evaluate and substantiate the validity of the hypothesis that certain specific anti-mycobacterial antibody responses are predictors of future active TB. If true, the reported low specificity of serodiagnostics for the detection of active TB would turn out to be due to a high risk for an early stage of progression to active disease and would then offer new opportunities to interrupt the cycle of transmission. If our findings are confirmed the clinical relevance would be as follows: a corresponding antibody-based test in endemic settings would then not differentiate between active TB cases on the one hand and latent or absent infection on the other hand. Instead, it would distinguish a high-risk group for preclinical or active TB from a group that is unlikely to progress to clinical disease. This would be of particular significance as such antibody tests could be developed into low-cost, point of care tests that may be used as screening tools, particularly in high TB burden low-resource settings. In such a clinical approach, the pre-screened high risk individuals could then be investigated using established assays such as X-ray, IGRA, sputum smear, sputum culture and GeneXpert MTB/RIF for final clinical assessment [3, 42]. Concerning the serodiagnostic results, those LTBI individuals showing the highest IgA or IgG antibody signals might have the highest risk to progress to active TB as their higher antibody levels may indicate higher bacterial loads. The detection of preclinical and early TB would have considerable clinical impact, as the identification, close monitoring, or early treatment of those LTBI with incipient disease would offer an opportunity to break the cycle of transmission and to prevent more serious lung destruction. In addition, early treatment of LTBI may decrease the emergence of multidrug resistance- (MDR-) TB strains [43], as the low numbers and slow turnover of mycobacteria in LTBI presumably present less opportunity to develop resistance.

In the present study the LTBI subject with the highest IgA levels against NarL, AlaDH and 19 kDa developed active TB within 3 months after recruitment. Although this is in line with the working hypothesis, one has to be very careful with its interpretation as we are dealing only with a single case. Clearly, as stated above, further research is needed to investigate whether antibody responses to mycobacterial antigens hold any prognostic significance for subsequent development of active TB in individuals with LTBI as previously suggested [4–6, 8].

Though a recent metaanalysis showed that so far neither IGRAs nor the TST can discriminate the ~90% of persons with true LTBI from the ~10% who will develop active TB [44], promising tendencies are noteworthy also in this field [45, 46]. Subjects with high risk for preclinical TB might be monitored more closely in defined time intervals using the currently best available resources and should be treated where applicable.

Due to the low frequency of reactivation in immunocompetent LTBI individuals even in TB endemic settings, large-scale longitudinal human studies would have to be conducted to further investigate whether serologic responses to mycobacterial antigens hold any prognostic significance for subsequent development of active TB in individuals with LTBI (correlates of risk). The results of this study and previous human and animal studies [4, 5, 7–9, 38–41] suggest that this is a promising approach, particularly as the serological tests could be performed on existing stored sera from previous studies. Our results suggest that for these investigations besides IgG also IgA serology should be considered. Due to the important role of IgA antibodies in the lung, the additional consideration of sputum IgA levels might be meaningful. The development of point-of-care tests that can be used as correlates of risk or as diagnostic for active TB would constitute a major advance.

## 5. Conclusions

In conclusion, as suggested in human studies and several animal models [4, 5, 7–9, 38–41], it remains a promising hypothesis that those latently infected individuals with antibody responses resembling those of active TB subjects are more prone to progression to active TB. Our finding of a nonrandom distribution of antibody responses among LTBI subjects suggests that the development of serodiagnostic kits for the determination of a high-risk group for preclinical or active TB in endemic settings may be possible. Furthermore, our results encourage the further investigation of IgA besides IgG responses in the field of serodiagnosis of active TB and possibly preclinical TB. Moreover, IgA antibodies against the novel antigen NarL were able to determine, besides the putative high-risk subgroup for preclinical TB (including highest levels for the actual progressor), the highest proportion of active TB patients.

## Supplementary Material

Supplementary Material and Methods:
1.1 Protein expression and purification.
1.2 Enzyme-linked immunosorbent assay
Supplementary Tables:
2.1 Table S1
2.2 Table S2

## Figures and Tables

**Table 1:** Recombinant antigens of *M. tuberculosis* used in this study.

Protein name(s)	Rv number	Mol mass (kDa)	Expression vector	6-fold His-Tag	*E. coli* host strain
NarL	Rv0844c	23.9	pET22	N-terminal	BL21 (DE3)
AlaDH	Rv2780	38.7	pJLA604	—	CAG629
19 kDa glycolipoprotein, LpqH	Rv3763	16.0	pET26	C-terminal	BL21 (DE3)
PstS3	Rv0928	38.8	pET22	C-terminal	Rosetta (DE3)
MPT83, MPB83	Rv2873	24.9	pET21	C-terminal	BL21 (DE3) [pLysS]

Nitrate/nitrite response transcriptional regulatory protein NarL; secreted L-alanine dehydrogenase (AlaDH); 19 kDa lipoprotein antigen precursor LpqH; periplasmic phosphate-binding lipoprotein PstS3; cell surface lipoprotein MPT83.

**Table 2:** Specificities and sensitivities of single seroantigens to distinguish between healthy non-TB infected controls [HC (*n* = 14)] and either active TB patients (*n* = 42) or LTBI [with medium or high IgA levels (*n* = 19)].

Antigen	Ig class	TB versus HC		LTBI (elevated IgA) versus HC	
		Sens. (%) (95% CI) based on 92.9% (95% CI, 66.1–99.8%) spec.	Sens. (%) (95% CI) based on 100% (95% CI, 76.8–100%) spec.	Sens. (%) (95% CI) based on 92.9% (95% CI, 66.1–99.8%) spec.	Sens. (%) (95% CI) based on 100% (95% CI, 76.8–100%) spec.
NarL	A	81.0 (65.9–91.4)^***^	78.6 (63.2–89.7)^***^	84.2 (60.4–96.6)^***^	84.2 (60.4–96.6)^***^
MPT83	A	47.6 (32.0–63.6)^##^	2.4 (0.06–12.6)^##^	63.2 (38.4–83.7)^###^	0 (0.0–17.6)^###^
19 kDa	A	64.3 (48.0–78.4)^***^	64.3 (48.0–78.4)^***^	78.9 (54.4–93.9)^***^	78.9 (54.4–93.9)^***^
PstS3	A	61.9 (45.6–76.4)^***^	35.7 (21.6–52.0)^***^	57.9 (33.5–79.7)^***^	31.6 (12.6–56.6)^***^
AlaDH	G	42.9 (27.7–59.0)^*^	28.6 (15.7–44.6)^*^	26.3 (9.1–51.2) n. s.	15.8 (3.4–39.6) n. s.
AlaDH	A	76.2 (60.5–87.9)^***^	69.0 (52.9–82.4)^***^	89.5 (66.9–98.7)^***^	84.2 (60.4–96.6)^***^

Significance levels of *P* values: *P* values refer to the *t*-test (if appropriate on log-transformed data) or the Mann-Whitney test where indicated. A *P* value of ≤0.05 was judged significant and levels of significance were indicated as follows: ^*^ or ^#^ *P* = 0.01–0.05, ^**^ or ^##^ *P* = 0.001–0.01, ^***^ or ^###^ *P* < 0.001; asterisks refer to parametric tests, hashes to nonparametric tests.

vs. = versus; n. s. = not significant; sens. = sensitivity; spec. = specificity.

The mean ranks of the IgA signals against the 5 investigated antigens were used to group the LTBI group into the top 19 groups [LTBI with elevated mean IgA levels (*n* = 19)] and the remaining LTBI group [LTBI (low IgA)].

The data were analyzed by using specificity levels of ≥90%, as described by other investigators [36, 37].

**Table 3:** Spearman correlation between the antibody performances for 53 individuals with LTBI^a^.

Anti-AlaDH IgG	Anti-AlaDH IgA	Anti-19 kDa IgA	Anti-PstS3 IgA	Anti-MPT83 IgA	
*r* = 0.17 (0.1–0.42) *P* = 0.22	*r* = 0.93 (0.87–0.96) *P* < 0.0001	*r* = 0.91 (0.85–0.95) *P* < 0.0001	*r* = 0.82 (0.71–0.89) *P* < 0.0001	*r* = 0.89 (0.81–0.93) *P* < 0.0001	anti-NarL IgA
—	*r* = 0.21 (−0.06–0.46) *P* = 0.13	*r* = 0.26 (−0.01–0.49) *P* = 0.064	*r* = 0.31 (0.04–0.53) *P* = 0.025	*r* = 0.27 (0.0–0.50) *P* = 0.052	anti-AlaDH IgG
—	—	*r* = 0.96 (0.93–0.98) *P* < 0.0001	*r* = 0.86 (0.77–0.92) *P* < 0.0001	*r* = 0.83 (0.72–0.90) *P* < 0.0001	anti-AlaDH IgA
—	—	—	*r* = 0.88 (0.80–0.93) *P* < 0.0001	*r* = 0.83 (0.73–0.90) *P* < 0.0001	anti-19 kDa IgA
—	—	—	—	*r* = 0.88 (0.80–0.93) *P* < 0.0001	anti-PstS3 IgA

^a^Spearman's coefficient *r* of rank correlation (95% CI for *r*).

## Abbreviations

TB: Tuberculosis
LTBI: Latent* Mycobacterium tuberculosis* infection
HC: Healthy controls.
