# Supplementary material for: A Subgroup of Latently Mycobacterium tuberculosis Infected Individuals Is Characterized by Consistently Elevated IgA Responses to Several Mycobacterial Antigens
Source: Mediators Inflamm. 2015 Aug 10;2015:364758. doi: 10.1155/2015/364758 (PMC4546975; doi:10.1155/2015/364758)
Supplement: Supplementary file 1 — Supplementary Material and Methods: 1.1 Protein expression and purification. 1.2 Enzyme-linked immunosorbent assay Supplementary Tables: 2.1 Table S1 2.2 Table S2 [file 364758.f1.pdf]

Supplements

§1. Material and methods

§1.1 Protein expression and purification

The purification of the antigens (besides the previously described purification of AlaDH) was performed as follows:

NarL: The cells were grown in LB and 400 µg/mL ampicillin at 37 °C overnight. The protein expression was induced with 0.2 mM IPTG. The cells were harvested by centrifugation, suspended in 20 mM tris/HCl, pH 8.0, and sonicated using a LABSONIC 2000 sonicator (B. Braun International). After centrifugation the supernatant was applied to a metal chelate affinity column. The fractions containing pure NarL protein as analysed by SDS PAGE were pooled. Densitometric SDS-PAGE analysis showed a purity of > 95 % for the final protein preparation.

19 kDa: The cells were grown in LB medium and 50 µg/mL kanamycin at 37 °C overnight. The protein expression was induced with 1 mM IPTG. The cells were harvested by centrifugation, suspended in 20 mM tris/HCl, 1 % Triton X-100, 100 mM NaCl pH 8.0, and disrupted using a LABSONIC 2000 sonicator. After centrifugation, the supernatant was applied to a metal chelate affinity column. The protein was eluted in fractions in a gradient of imidazole. The fractions containing pure 19 kDa protein as analysed by SDS PAGE were pooled. Purity analysis by automated electrophoresis with fluorimetric detection and densitometric purity measurement showed a purity of > 95 % for the final protein preparation.

PstS3: For production of PstS3, cells were grown in LB medium at 37° C, induced with 1 mM IPTG and incubated at 37° C for further 4 h. After centrifugation, the cells were suspended in 50 mM potassium phosphate buffer, pH 7.3. The cells were sonicated using a Bandelin GM70 sonifyer. After

centrifugation, the pellet was solubilized in buffer containing 8 M urea. The protein was purified on a metal chelate affinity column. The protein solution was refolded by buffer exchange on a gel filtration column. The protein containing high molecular weight fraction of this gel filtration step was collected and subjected to another affinity purification step on a metal chelate affinity column. Elution was done using 500 mM imidazole. The fractions containing pure PstS3 protein were pooled. Purity analysis by automated electrophoresis with fluorimetric detection and densitometric purity measurement showed a purity of > 95 % for the final protein preparation.

MPT83: The cells were grown in Terrific Broth medium containing 400 µg/mL ampicillin and 34 µg/mL chloramphenicol overnight. The protein expression was induced with 0.2 mM IPTG. After further 4.5 h of incubation, the cells were harvested by centrifugation and suspended in BugBusterTM Protein Extraction Reagent (Novagen). After centrifugation, the pellet was resuspended in 20 mM tris/HCl, 150 mM NaCl, pH 8.0 containing 1 % Triton X-100. The suspension was homogenized using a Miccra D-8 homogenizer. After a washing step, the pellet was solubilized using 8 M urea as denaturation agent. MPB83 was refolded on a gel filtration column. The refolded protein was bound to an anion exchange column and eluted in a salt gradient from 0-500 mM NaCl. The fractions containing pure MPB83 protein as analysed by SDS PAGE were pooled. Purity analysis by automated electrophoresis with fluorimetric detection and densitometric purity measurement showed a purity of > 95 % for the final protein preparation.

§1.2 Enzyme-linked immunosorbent assay

The antigens were diluted in 0.15 M PBS, pH 7.5 (concentration (measured by Lowry based assay) = 1µg antigen/ml coating buffer). 100 µl of the coating solution (antigen in coating buffer) was pipetted into each well of the microtiter plates (Greiner, Maxisorb). The microtiter plates were incubated over night at 2-8°C. The following day the plates were washed 3x with wash solution (0.15

M PBS, pH 7.5 + 0.05 % Tween-20, 300 µl/well). 300 µl of blocking solution (0.15 M PBS, pH 7.5 + 1 % BSA) were pipetted into each well of the coated plates and the plates were incubated for 2 h at 37°C. Finally, the plates were washed again 3 times with the wash buffer. Diluted serum samples from human (diluted 1:200 in 0.15 M PBS, pH 7.5 + 0.5 % BSA, 100 µl/well) were pipetted into the wells of the antigen coated microtiter plate. After 45 minutes incubation at 37°C, the washing step was repeated, in order to remove unbound material. Then ready-to-use peroxidase conjugate was added (100 µl/well) and incubated for 30 minutes at 37°C. For the detection of IgG antibodies anti-human-IgG (Fc)-HRP (Pierce, diluted 1:40,000 in blocking solution) was used. Specific IgA antibodies were detected with anti-human-IgA (a)-HRP (Pierce, diluted 1:12,000 in blocking solution). After a further washing step, the substrate tetramethylbenzidine (TMB) solution was pipetted (100 µl/well) and incubated for 10 minutes at 37°C. The colour development was terminated by the addition of 100 µl of stop solution (0.2 M sulphuric acid). The resulting dye was measured by an automatic microplate reader (MR7000; Dynatech Deutschland GmbH, 73274 Notzingen, Germany) at the wavelength of 450 nm with the reference wavelength 620 nm. Positive and negative control sera as well as blanks were included at least in duplicate to control for inter- and intra-run variations. If duplicate values differed by >15%, the measurement was repeated.

§2. Supplementary tables

§2.1

TABLE S1. Specificities and sensitivities of single seroantigens to distinguish between healthy non-TB infected controls [HC* (n=12)] and either active TB patients (n=42) or LTBI [with medium or high IgA levels (n=19)]

[*Table S1 represents an alternative calculation to Table 2. The only difference is that both IGRA-negative individuals with Mantoux indurations between 5 and 15 mm were transferred from the HC group (HC*: n=12) into the LTBI group.*]

Antigen	Ig class	TB vs. HC*		LTBI (elevated IgA) vs. HC*	
		Sens. (%) (95% CI) based on 91.7% (95% CI, 61.5–99.8%) spec.	Sens. (%) (95% CI) based on 100% (95% CI, 73.5–100%) spec.	Sens. (%) (95% CI) based on 91.7% (95% CI, 61.5–99.8%) spec.	Sens. (%) (95% CI) based on 100% (95% CI, 73.5–100%) spec.
NarL	A	81.0 (65.9–91.4) ***	78.6 (63.2–89.7) ***	84.2 (60.4–96.6) ***	84.2 (60.4–96.6) ***
MPT83	A	47.6 (32.0–63.6) #	2.4 (0.06–12.6) #	63.2 (38.4–83.7) ###	0 (0.0–17.6) ###
19 kDa	A	64.3 (48.0–78.4) ***	64.3 (48.0–78.4) ***	78.9 (54.4–93.9) ***	78.9 (54.4–93.9) ***
PstS3	A	61.9 (45.6–76.4) ***	35.7 (21.6–52.0) ***	57.9 (33.5–79.7) ***	31.6 (12.6–56.6) ***
AlaDH	G	42.9 (27.7–59.0) n. s.	28.6 (15.7–44.6) n. s.	26.3 (9.1–51.2) n. s.	15.8 (3.4–39.6) n. s.
AlaDH	A	76.2 (60.5–87.9) ***	69.0 (52.9–82.4) ***	89.5 (66.9–98.7) ***	84.2(60.4–96.6) ***

Significance levels of P-values: P-values refer to the t-test (if appropriate on log-transformed data) or the Mann-Whitney test where indicated. A p-value of ≤ 0.05 was judged significant and levels of significance were indicated as follows: * or #: p = 0.01–0.05, ** or ##: p = 0.001–0.01, *** or ###: p < 0.001; asterisks refer to parametric tests, hashes to non-parametric tests.

vs. = versus; n. s. = not significant; sens. = sensitivity; spec. = specificity.

The mean ranks of the IgA signals against the 5 investigated antigens were used to group the LTBI group into the top 19 group [LTBI with elevated mean IgA levels (n=19)] and the remaining LTBI group [LTBI (low IgA)].

§2.2

TABLE S2. Spearman correlation between the antibody performances for individuals with latent TB (n=55; LTBI*) [a]

[*Table S2 represents an alternative calculation to Table 3. The only difference is that both IGRA-negative individuals with Mantoux indurations between 5 and 15 mm were added to the LTBI group (n=53) described in Table 3.*]

anti-AlaDH IgG	anti-AlaDH IgA	anti-19 kDa IgA	anti-PstS3 IgA	anti-MPT83 IgA	
r = 0.18 (-0.09–0.43) P = 0.19	r = 0.93 (0.87–0.96) P < 0.0001	r = 0.91 (0.85–0.95) P < 0.0001	r = 0.82 (0.71–0.89) P < 0.0001	r = 0.89 (0.82–0.93) P < 0.0001	anti-NarL IgA
[illegible]	r = 0.22 (-0.04–0.46) P = 0.10	r = 0.26 (0.0–0.49) P = 0.054	r = 0.31 (0.05–0.53) P = 0.020	r = 0.29 (0.03–0.52) P = 0.032	anti-AlaDH IgG

		r = 0.96 (0.94–0.98)<br>$P < 0.0001$	r = 0.86 (0.78–0.92)<br>$P < 0.0001$	r = 0.83 (0.73–0.90)<br>$P < 0.0001$	anti-AlaDH IgA
			r = 0.88 (0.80–0.93)<br>$P < 0.0001$	r = 0.83 (0.73–0.90)<br>$P < 0.0001$	anti-19 kDa IgA
				r = 0.88 (0.80–0.93)<br>$P < 0.0001$	anti-PstS3 IgA

[a] Spearman's coefficient r of rank correlation (95% CI for r)
